# Expression Patterns and Levels of All Tubulin Isotypes Analyzed in GFP Knock-In *C. elegans* Strains

**DOI:** 10.1247/csf.21022

**Published:** 2021-05-08

**Authors:** Kei Nishida, Kenta Tsuchiya, Hiroyuki Obinata, Shizuka Onodera, Yu Honda, Yen-Cheng Lai, Nami Haruta, Asako Sugimoto

**Affiliations:** 1 Laboratory of Developmental Dynamics, Graduate School of Life Sciences, Tohoku University, Sendai 980-8577, Japan

**Keywords:** tubulin isotypes, microtubules, *C. elegans*

## Abstract

Most organisms have multiple α- and β-tubulin isotypes that likely contribute to the diversity of microtubule (MT) functions. To understand the functional differences of tubulin isotypes in *Caenorhabditis elegans*, which has nine α-tubulin isotypes and six β-tubulin isotypes, we systematically constructed null mutants and GFP-fusion strains for all tubulin isotypes with the CRISPR/Cas9 system and analyzed their expression patterns and levels in adult hermaphrodites. Four isotypes—α-tubulins TBA-1 and TBA-2 and β-tubulins TBB-1 and TBB-2—were expressed in virtually all tissues, with a distinct tissue-specific spectrum. Other isotypes were expressed in specific tissues or cell types at significantly lower levels than the broadly expressed isotypes. Four isotypes (TBA-5, TBA-6, TBA-9, and TBB-4) were expressed in different subsets of ciliated sensory neurons, and TBB-4 was inefficiently incorporated into mitotic spindle MTs. Taken together, we propose that MTs in *C. elegans* are mainly composed of four broadly expressed tubulin isotypes and that incorporation of a small amount of tissue-specific isotypes may contribute to tissue-specific MT properties. These newly constructed strains will be useful for further elucidating the distinct roles of tubulin isotypes.

## Introduction

Microtubules (MTs) play various essential roles in cell division, ciliogenesis, cell polarity, and intracellular transportation (Desai and Mitchison, 1997). MTs are hollow cylindrical structures composed of α- and β-tubulin heterodimers (Amos and Klug, 1974). In most organisms, both α- and β-tubulin are encoded at multiple genomic loci. As proposed in the ‘multi-tubulin hypothesis’ over 40 years ago (Fulton and Simpson, 1976), different tubulin isotypes are believed to contribute to the diversity of MT functions. For example, in humans, which have nine α-tubulin and nine β-tubulin isotypes, mutations in tubulin isotypes cause different types of diseases; mutations in the α-tubulin isotype TUBA3 cause microcephaly or cerebral hypertrophy (Keays *et al.*, 2007), and mutations in β-tubulin isotype TUBB3 cause CFEOM3 (congenital fibrosis of the extraocular muscles type 3) (Tischfield *et al.*, 2010). The recent accumulation of expression data also suggests that each tubulin isotype has different expression patterns with partial overlap among isotype expression (Leandro-García *et al.*, 2010; Lewis *et al.*, 1985; Hurd, 2018) and that multiple tubulin isotypes co-exist within a cell and cooperatively generate tissue-specific MTs (Leandro-García *et al.*, 2010; Lewis *et al.*, 1985). However, how each tubulin isotype contributes to the functional diversity of MTs remains largely unknown, because it has been challenging to observe MT dynamics *in vivo* in most organisms.

*Caenorhabditis elegans* is a suitable model organism for examining the role of each tubulin isotype *in vivo*. Its transparent and simple body plan and the sophisticated tools available to manipulate this species genetically enable the live imaging analysis of MT behaviors. *C. elegans* has nine α- and six β-tubulin isotypes, and some tubulin isotypes have been genetically shown to have tissue-specific functions. For example, mechanosensory neuron-specific α-tubulin *mec-12* and β-tubulin *mec-7* were identified as touch-insensitive mutants (Chalfie and Sulston, 1981; Chalfie and Au, 1989). Moreover, ciliated neuron-specific α-tubulin isotype *tba-6* is necessary for the typical configuration of MTs in cilia (Silva *et al.*, 2017).

The tissue- or stage-specific expression patterns of tubulin isotypes were analyzed here by several methods. In *C. elegans*, transgenes that express fluorescent protein-tagged tubulins have been used either as extrachromosomal arrays or as additional copies integrated into the genome, but endogenous expression levels are often not precisely represented with these approaches (Hurd *et al.*, 2010; Hao *et al.*, 2011). In mice, immunostaining using isotype-specific antibodies has been used, but the availability of specific antibodies that distinguish each isotype is limited because of the highly conserved nature of tubulin structures (Hausrat *et al.*, 2020). Therefore, precise information regarding expression levels and the functional contribution of each tubulin isotype is still limited.

Previously we constructed site-specific and null mutant strains and GFP knock-in strains for four tubulin isotypes expressed in the early *C. elegans* embryos (TBA-1, TBA-2, TBB-1, and TBB-2) using the CRISPR/Cas9 system (Honda *et al.*, 2017). This manipulation of endogenous tubulin genes enabled the quantitative evaluation of how the concentration and ratio of the isotypes affect MT dynamics in early embryos and revealed that these four tubulin isotypes have different expression levels and distinct contributions to MT dynamics (Honda *et al.*, 2017).

In this study, we expanded this approach and constructed null mutants and GFP knock-in strains by CRISPR/Cas9-based genome editing and comprehensively and quantitatively analyzed the tissue-specific and stage-specific expression patterns of all tubulin isotypes in adult hermaphrodites of *C. elegans*. We found that the broadly expressed isotypes were expressed at significantly higher levels than tissue-specific isotypes and that one neuron-specific β-tubulin isotype was inefficiently incorporated into mitotic spindle MTs.

## Results

### Construction of GFP knock-in strains for all tubulin isotypes

To understand the distinct functions and expression patterns of the nine α-tubulin and six β-tubulin isotypes of *C. elegans*, we systematically constructed individual null mutants that express GFP from each of the tubulin gene promoters and strains in which the endogenous isotype proteins are tagged with GFP with the CRISPR/Cas9-mediated two-step system (Dickinson *et al.*, 2015). For *tba-1*, *tba-2*, *tbb-1*, and *tbb-2*, corresponding strains were previously constructed (Honda *et al.*, 2017). The GFP-coding fragment was knocked in at the N terminus of each tubulin protein, as the C terminus interacts with various microtubule-associated proteins (MAPs) and motor proteins and has multiple sites that undergo post-translational modifications (PTMs) (Skiniotis *et al.*, 2004; Hallak *et al.*, 1977; Arce *et al.*, 1975; L’Hernault and Rosenbaum, 1985).

In the first step, null mutants were constructed by inserting the fragment containing the GFP-encoding gene and a self-excising cassette (SEC; containing *loxP* excision sites, the Roller phenotype-causing *sqt-1(d)* gene, the gene encoding Cre recombinase, and the hygromycin B resistance gene) in the N terminus of the coding region of an individual α- or β-tubulin gene (Dickinson *et al.*, 2015). These strains thus express GFP under the control of a tubulin promoter, but the tubulin-encoding regions cannot be transcribed because a transcriptional terminator is contained within the SEC (Dickinson *et al.*, 2015). All 13 GFP-SEC knock-in strains constructed in this study were viable (Table I), consistent with previous reports on tubulin isotype mutants, which concluded that only TBB-2 is essential for viability (see references in Table I). As these strains show the Roller phenotype due to *sqt-1(d)* in the SEC, behavioral analyses were not carried out.

In the second step, GFP translational fusion strains were constructed by removing SEC by heat shock treatments. In these strains, endogenous tubulin isotypes were labeled with GFP at their N termini, and their expression patterns and subcellular localization could then be analyzed. The GFP signals in these genome-edited strains are expected to represent the quantities of the endogenous tubulin isotype proteins more precisely than in the previously reported tubulin reporter strains constructed with exogenous copies (Hurd *et al.*, 2010; Hurd, 2018; Hao *et al.*, 2011)

### Expression patterns of tubulin isotypes in adult hermaphrodites

**Fig. 1:**
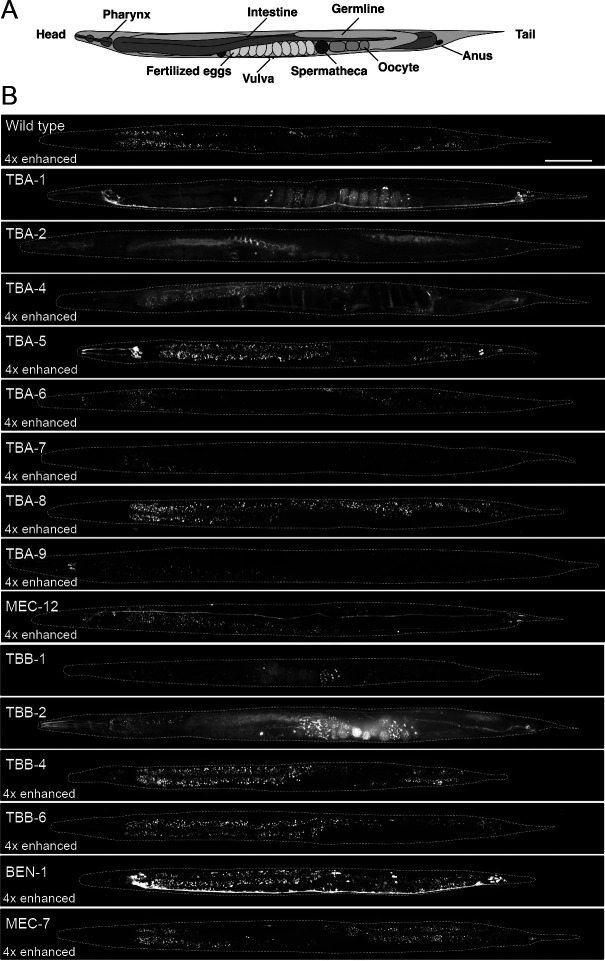
Expression patterns of all tubulin isotypes in *C. elegans* adult hermaphrodites. (A) Schematic drawing of a *C. elegans* adult hermaphrodite. (B) The whole body of adult hermaphrodites expressing GFP-tagged tubulin isotypes. Images except for those of *gfp::tba-1*, *gfp::tba-2*, *gfp::tbb-1*, and *gfp::tbb-2* were enhanced four times. White dashed lines indicate the outline of the body of each worm. Scale bar: 100 μm. All images were arranged such that the head is at the left and the ventral side is at the bottom, except for the images of *tba-9* and *mec-12*, which were acquired from the ventral side. Images without enhancement are shown in Fig. S1.

Using our comprehensive collection of GFP knock-in strains in this study and ones made in a previous study (Honda *et al.*, 2017), we re-evaluated the tissue specificity and expression level for each tubulin isotype in the adult hermaphrodite by confocal fluorescence microscopy (Fig. 1).

**Fig. 2:**
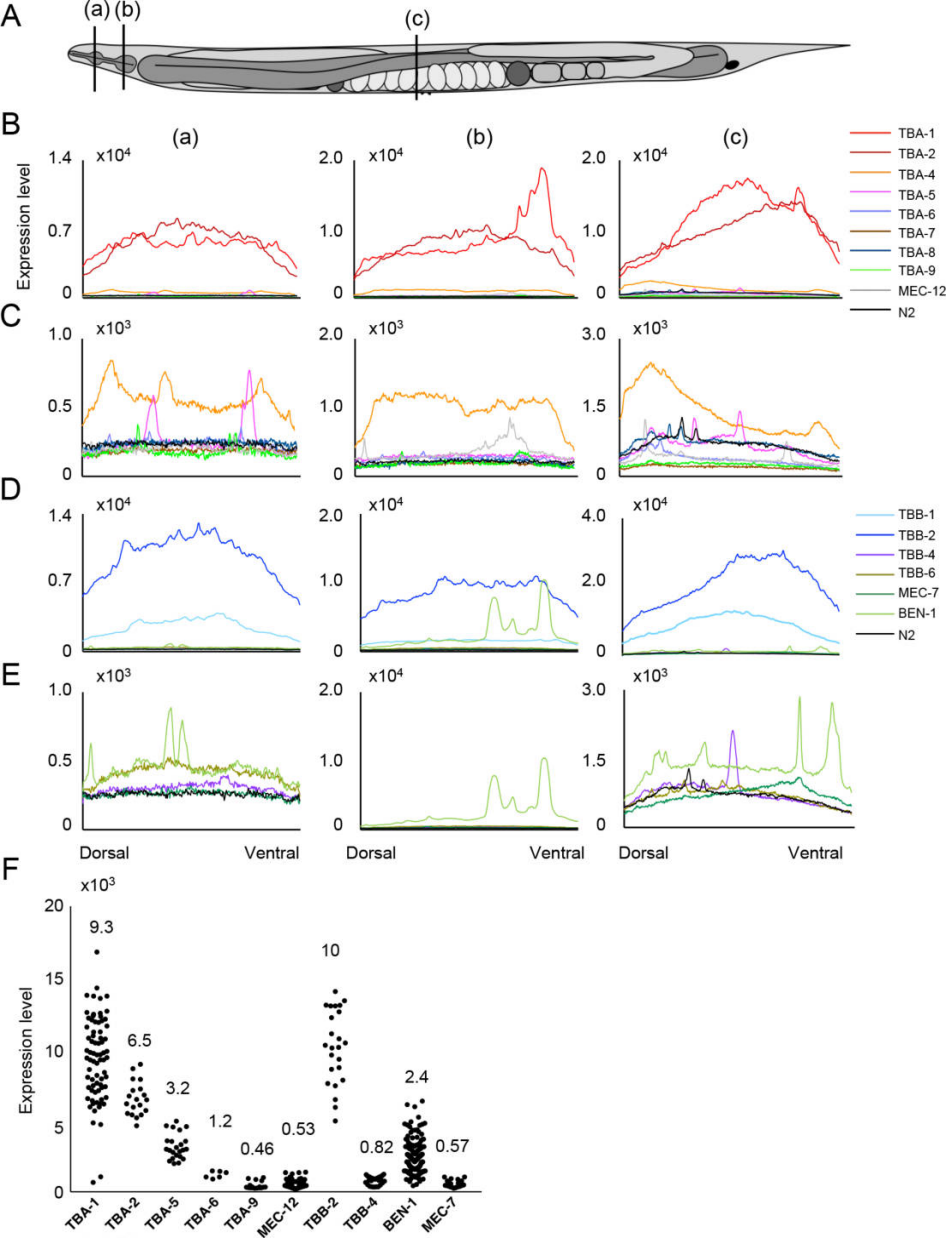
Comparison of expression levels for all tubulin isotypes in *C. elegans* adult hermaphrodites. (A) Schematic drawing of the whole body of a *C. elegans* adult hermaphrodite. The lines (a), (b), and (c) indicate the sections where the GFP intensities were analyzed: (a) metacorpus region, (b) posterior bulb region, and (c) the anterior region of the vulva. (B–E) The signal intensities of each isotype at the sections indicated in (A). (B) All α-tubulin isotypes. (C) Tissue-specific α-tubulin isotypes. (D) All β-tubulin isotypes. (E) Tissue-specific β-tubulin isotypes. (F) The signal intensity of tubulin expression in cell bodies of neuronal cells. TBB-1 is not included because it was not detected in cell bodies. Each dot indicates the signal intensity in a single cell body, and all detectable cells were measured. The average intensity for each isotype was determined as described in the Methods and is shown for each isotype.

The expressed tissues or cell types observed in this study were generally consistent with previous studies that used multi-copy/extrachromosomal transgene expression (Hurd *et al.*, 2010; Hurd, 2018; Hao *et al.*, 2011). The expression levels of tubulin isotypes were compared based on their GFP intensity in cross-sections of the head (a, metacorpus; b, posterior bulb) and the middle body region (c, the anterior region of the vulva) of the adult hermaphrodites (Fig. 2A). We found that four broadly expressed isotypes (TBA-1, TBA-2, TBB-1, and TBB-2) were significantly expressed at higher levels than other isotypes expressed in specific tissues (Fig. 1B and Fig. 2B, D). Among the tissue-specific isotypes, four α-tubulin isotypes (TBA-5, TBA-6, TBA-9, and MEC-12) and three β-tubulin isotypes (TBB-4, MEC-7, and BEN-1) were specifically expressed in neurons, and the remaining isotypes (TBA-4, TBA-7, TBA-8, and TBB-6) were expressed in other tissues (Fig. 1B), whose details are described below.

### Expression of broadly expressed tubulin isotypes in adult hermaphrodites

**Fig. 3:**
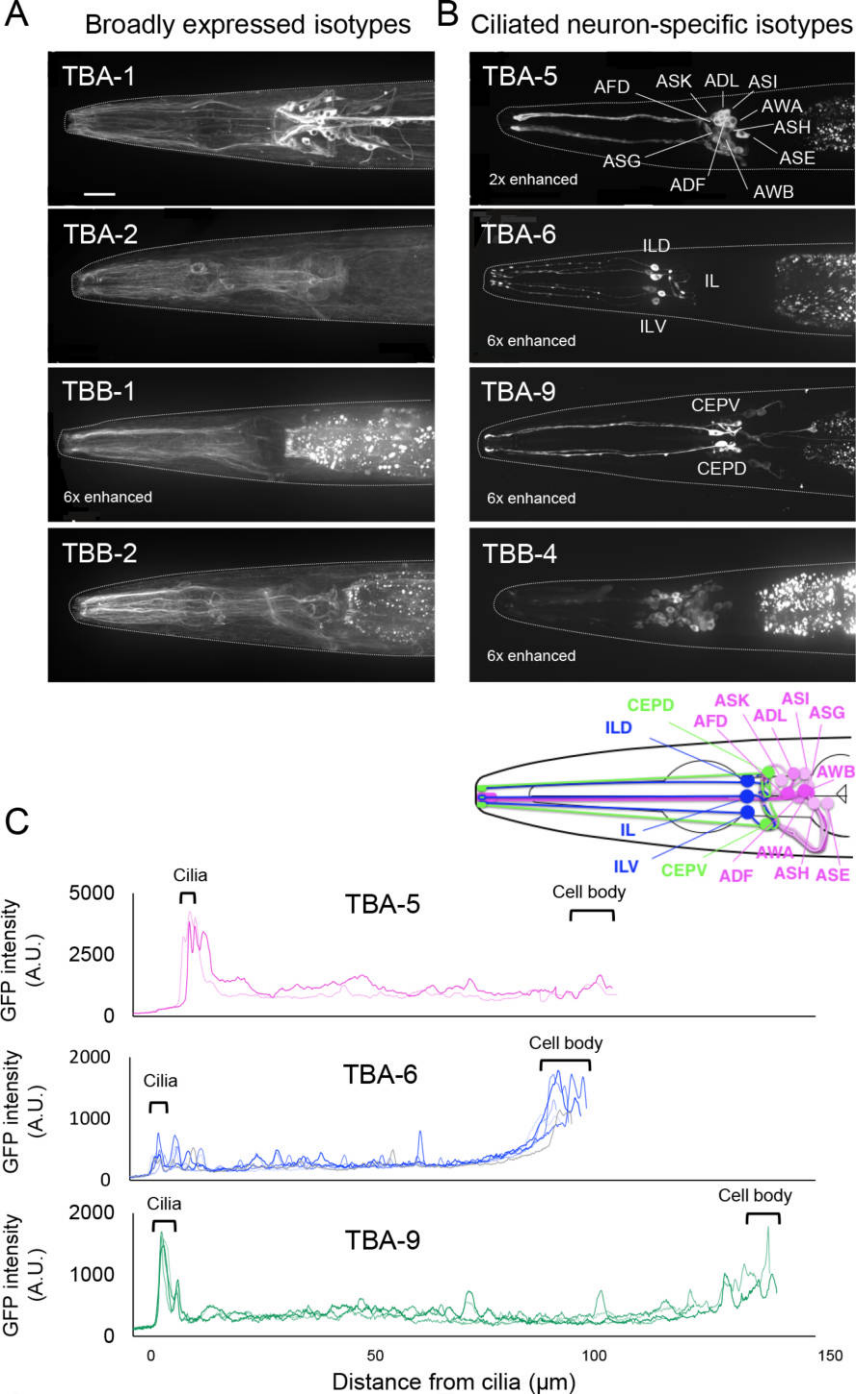
Ciliated sensory neurons express distinct sets of tubulin isotypes. (A, B) Fluorescence images of head regions of adult hermaphrodites. Scale bar: 25 μm. The images except for those of *gfp::tba-1*, *gfp::tba-2*, and *gfp::tbb-2* were enhanced as indicated. (A) Broadly expressed isotypes (TBA-1, TBA-2, TBB-1, and TBB-2). (B) Ciliated neuron-specific isotypes (TBA-5, TBA-6, TBA-9, and TBB-4). The bottom diagram summarizes the sets of sensory neurons that express TBA-5, TBA-6, and TBA-9. Magenta: TBA-5, blue: TBA-6, green: TBA-9. The TBA-6-expressing neurons are either IL1 or IL2, which could not be distinguished. (C) GFP intensities for TBA-5, TBA-6, and TBA-9 along the ciliated neurons. The GFP intensities were tracked from 5 μm outside the neuronal ending to the end of the cell body. Each line indicates the measurements from a single neuronal cell, and cells from a single worm are shown. Brackets indicate the regions corresponding to the cilia and cell body.

TBA-1, TBA-2, TBB-1, and TBB-2 were broadly expressed in virtually all tissues, although the specific patterns of their expression differed (Fig. 1B and Fig. 3A). The α-tubulins TBA-1 and TBA-2 showed similar expression levels in adult hermaphrodites as a whole, based on the signal intensities of cross-sections in the head and middle body region (Fig. 2B), but their tissue specificities were different: TBA-1 was expressed primarily in neurons and the germline, whereas TBA-2 was expressed at higher levels in the pharynx and intestine than in other tissues (Fig. 1B and Fig. 3A). More neuronal cells expressed TBA-1 than TBA-2, and quantification of the GFP intensity of each neuronal cell body showed that the average signal intensity of TBA-1 was ~1.4 times higher than that of TBA-2 (Fig. 2F).

Although two β-tubulins, TBB-1 and TBB-2, were expressed in almost all tissues, TBB-2 was expressed at higher levels than TBB-1: ~4.5 times higher in the head and ~2.4 times higher in the middle body region than TBB-1, based on the signal intensity of cross-sections (Fig. 2D and Fig. S1B). Within neurons, both TBB-1 and TBB-2 were detected in axons at a higher level than in cell bodies, whereas TBA-1 was present in both axons and cell bodies at comparable levels (Fig. 3A).

### Expression of ciliated neuron-specific tubulin isotypes in adult hermaphrodites and during embryogenesis

TBA-5, TBA-6, TBA-9, and TBB-4 were expressed in ciliated sensory neurons (Fig. 1B and Fig. 3B), in which cilia are present at the ends of their dendritic processes. Whereas TBA-5, TBA-6, and TBA-9 were detected in small subsets of these neurons, TBB-4 was expressed in most ciliated sensory neurons. TBA-5 was expressed in amphid neurons (ADF, ADL, AFD, ASE, ASG, ASH, ASI, ASK, AWA, and AWB) and phasmid neurons (PHA and PHB), consistent with previous reports (Hao *et al.*, 2011; Bargmann, 2006) (Fig. 3B). In cell bodies, the average expression level of TBA-5 was ~30% of that of TBA-1 (Fig. 2F). TBA-6 was detected in six neurons that likely correspond to ciliated neurons either IL1 (IL1, IL1D, and IL1V) or IL2 (IL2, IL2D, and IL2V) (Fig. 3B). Previously, TBA-6 was reported to be expressed in HSN and IL2 neurons (Hurd *et al.*, 2010), but HSNs were not detected in our strain. In cell bodies, the average expression level of TBA-6 was ~10% of that of TBA-1 (Fig. 2F). TBA-9 was detected in ciliated sensory neurons in the head (CEPV and CEPD) and in the ventral motor neuron PDE, as described previously (Hurd *et al.*, 2010). TBA-9 was expressed in an additional 13 neuronal cells in the head region whose identity was unable to be determined due to their low GFP signals (Fig. 3B) but might include the ones reported in Hurd *et al.* (2010), i.e., ADF, AFD, ASE, ASI, AWA, AWC, and ADE. The average expression of TBA-9 was ~5% of that of TBA-1 in the cell bodies (Fig. 2F). TBB-4 was expressed in many ciliated sensory neurons, consistent with a previous report (Hurd *et al.*, 2010). TBB-4 accumulated mainly in the cell body, and weak signals were detected in axons and cilia (Fig. 3B). In cell bodies, the average expression of TBB-4 was ~8% of that of TBB-2 (Fig. 2F).

Signal quantification in the cross-section that included the dendritic region (Fig. 2A, (a)) indicated that the levels of TBA-5, TBA-6, and TBA-9 were ~5% of those of the broadly expressed α-tubulin isotypes (TBA-1 and TBA-2) (Fig. 2B, C), and the level of TBB-4 was ~10% of those of the broadly expressed β-tubulin isotype TBB-2 (Fig. 2D). Within the ciliated neurons, TBA-5 accumulated in the region of the cilia, and TBA-9 was enriched in both cilia and cell bodies. TBA-6 was slightly enriched in the cilia and was detectable in cell bodies (Fig. 3C).

**Fig. 4:**
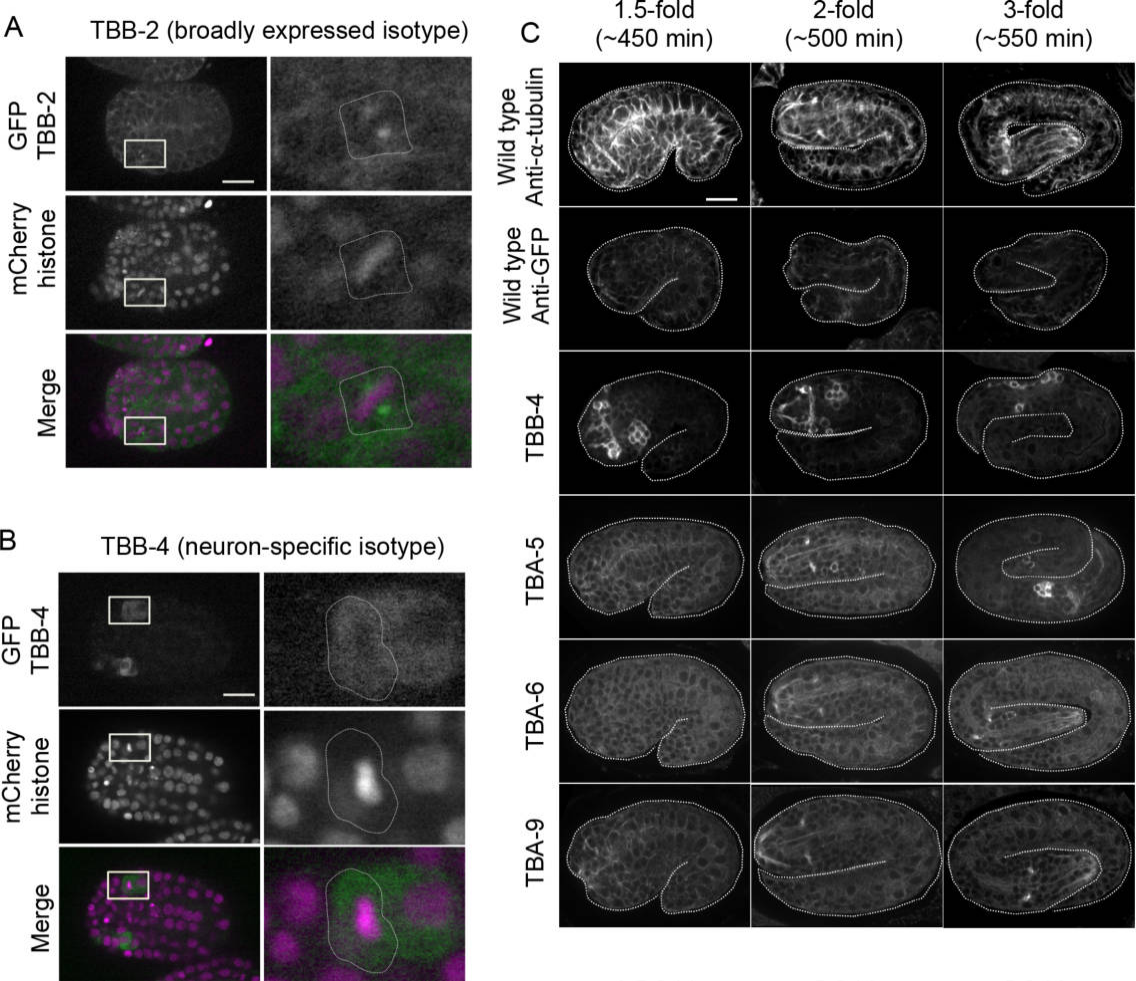
Embryonic expression of ciliated neuron-specific tubulin isotypes. (A, B) Expression and subcellular localization of GFP::TBB-2 and GFP::TBB-4 during the morphogenesis stage of embryogenesis (~300 min after fertilization). (A) An embryo that expresses GFP::TBB-2 and mCherry::Histone. (B) An embryo that expresses GFP::TBB-4 and mCherry::Histone. Scale bar in A and B: 10 μm. The right panels are magnified images from the white boxed regions shown in the left panels. White dashed lines indicate the outline of a metaphase cell. (C) Expression of ciliated neuron-specific tubulin isotypes during mid-to-late embryogenesis. Immunofluorescent images of the wild-type, and strains that express GFP::TBB-4, GFP::TBA-5, GFP::TBA-6, and GFP::TBA-9 at the indicated stages of development. Top row: staining wild type embryo with anti-α-tubulin antibodies to stain all microtubules. Other rows: staining with anti-GFP antibodies, to stain GFP-tagged isotypes. Scale bar in C: 10 μm.

To determine at which stage these ciliated neuron-specific α-tubulin isotypes (TBA-5, TBA-6, and TBA-9) and β-tubulin isotype TBB-4 initiate co-expression, we examined their expression during embryogenesis. TBB-4 was detectable in the precursor cells of ciliated sensory neurons around the dorsal enclosure stage (~300 min after fertilization) (Fig. 4B). At this stage, the broadly expressed isotype TBB-2 was assembled into spindle MTs in mitotic cells, but TBB-4 was not detected in the spindle MTs and instead was diffusely present in the cytoplasm (Fig. 4A, B), suggesting that TBB-4 was not efficiently incorporated into spindle MTs. TBB-4 was strongly expressed in the cell body of ciliated neuron precursors in 1.5-fold embryos (~450 min after fertilization). In contrast, ciliated neuron-specific α-tubulin isotypes (TBA-5, TBA-6, and TBA-9) became detectable during the 2-fold stage (~500 min after fertilization), and their accumulation in the tips of neurons, which correspond to the region of the cilia, became prominent at the 3-fold stage (~550 min after fertilization) (Fig. 4C).

Based on these expression patterns, we speculate that TBB-4 may contribute to the general MT properties of ciliated sensory neurons, whereas expression of additional tubulins TBA-5, TBA-6, and TBA-9 may confer some MT features specific to the subtype of ciliated neurons.

### Expression of other neuron-specific tubulin isotypes in adult hermaphrodites

**Fig. 5:**
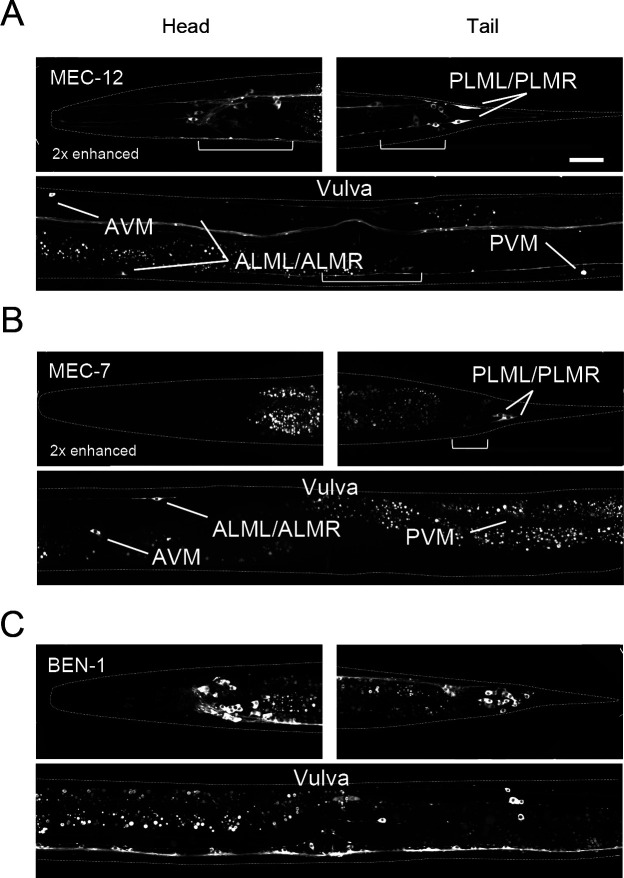
Expression of neuron-specific isotypes, MEC-7, MEC-12, and BEN-1. (A–C) Fluorescence images of head (upper left), tail (upper right), and around the vulva (bottom) of adult hermaphrodites for (A) gfp::mec-12, (B) gfp::mec-7 (enhanced as indicated), and (C) gfp::ben-1 strains. White dashed lines indicate the outlines of worm bodies. Mechanosensory neurons (ALML/ALMR, AVM, PLML/PLMR, and PVM) are indicated in mec-7 and mec-12. The white brackets indicate non-mechanosensory neuronal cells expressing MEC-7 or MEC-12. The images of GFP::mec-7 and GFP::mec-12 were enhanced two times. Scale bar in A: 25 μm (in A–C).

The α-tubulin MEC-12 and the β-tubulin MEC-7 are expressed in six mechanosensory neurons (ALML/ALMR, AVM, PLML/PLMR, and PVM) (Fukushige *et al.*, 1999; Hamelin *et al.*, 1992; Mitani *et al.*, 1993). In the *gfp::mec-12* strain, we detected MEC-12 expression in these same six mechanosensory neurons (Fukushige *et al.*, 1999) and in an additional ~90 unidentified neurons (Fig. 5A), which may overlap with the additional cells referred to previously (Fukushige *et al.*, 1999). In the *gfp::mec-7* strain, we detected the MEC-7 signal in these six mechanosensory neurons as reported (Hamelin *et al.*, 1992; Mitani *et al.*, 1993), as well as in an additional 16 neurons (Fig. 5B). In all detectable cells, the expression of MEC-12 and of MEC-7 in cell bodies was comparable and was ~6 % of that of the corresponding broadly expressed α-tubulin and β-tubulin isotype, respectively (Fig. 2F).

BEN-1 was expressed in various types of neuronal cells, including mechanosensory neurons (Fig. 1 and Fig. 5C). The average expression level of BEN-1 in cell bodies was three to four times higher than that of the neuron-specific β-tubulin isotypes TBB-4 and MEC-7 but was ~20% of that of the broadly expressed β-tubulin isotype TBB-2 (Fig. 2F).

Thus, in neuronal cells, the broadly expressed isotypes (TBA-1, TBA-2, TBB-1, and TBB-2) are expressed at a high level, and neuronal-specific isotypes are additionally expressed at a low level and may confer cell-type-specific MT features.

### Expression of other tubulin isotypes in adult hermaphrodites

**Fig. 6:**
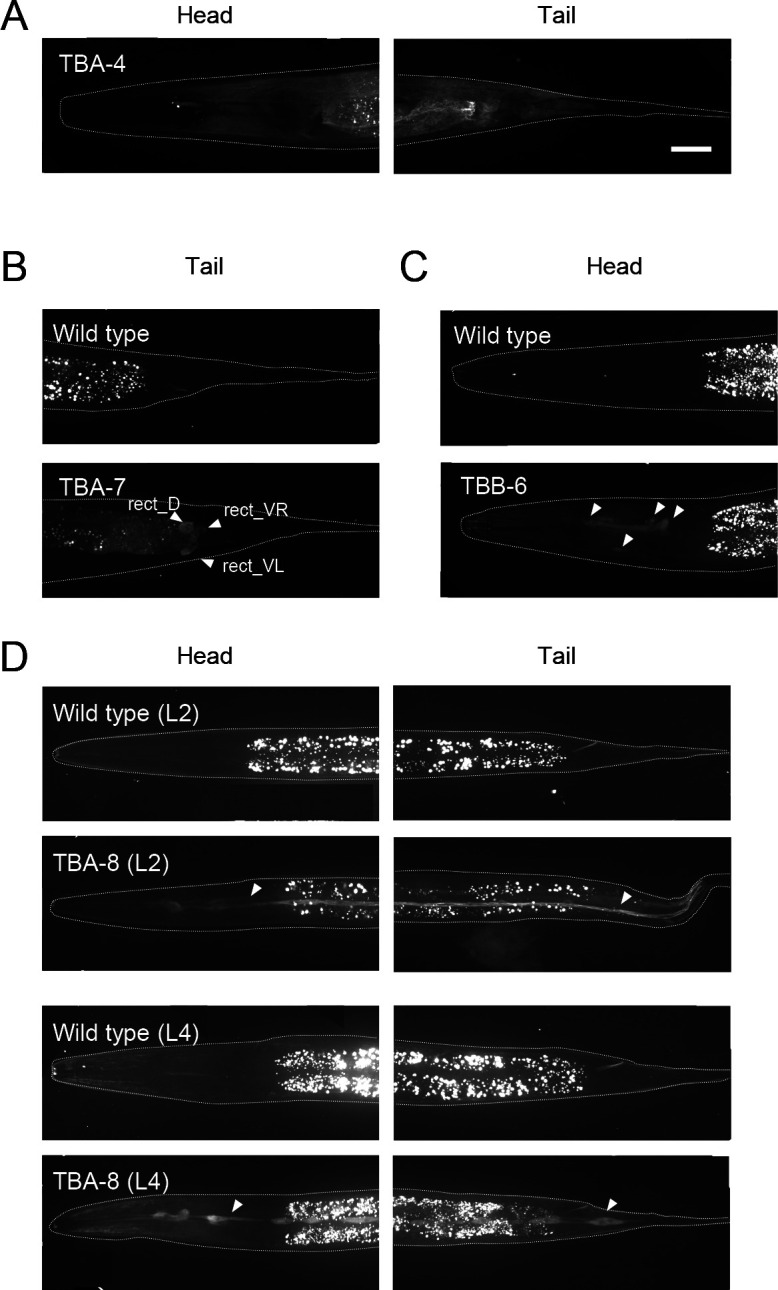
Expression of tissue-specific isotypes TBA-4, TBA-7, TBB-6, and TBA-8. (A) TBA-4 expression in the head and tail regions. (B) TBA-7 expression in several cells in the tail region. Top: wild type; bottom: GFP::TBA-7. White arrowheads indicate rect_D and rect_VL/R cells, which express TBA-7 and are involved in excretion. (C) TBB-6 expression in several cells in the head region whose identities are unclear (white arrowheads). Top: wild type; bottom: GFP::TBB-6. (D) TBA-8 expression in larval seam cells. Images of head and tail regions of second larval stage (L2) and fourth larval stage (L4) larvae are shown. Top: wild type; bottom: GFP::TBB-8. White arrowheads indicate seam cells. White dashed lines indicate the outline of the worm body in A–D. Scale bar in A: 25 μm (in A–D).

The isotypes TBA-4, TBA-7, TBA-8, and TBB-6 were expressed in distinct tissues. TBA-4 was expressed in a wide range of tissues, including the intestine and epidermis, and a small number of neurons but not in the germline (Fig. 1B and Fig. 6A). The general expression of TBA-4 was much lower than that of the broadly expressed isotypes TBA-1 and TBA-2 (~10% in section (a), (b), and (c) in Fig. 2). TBA-7 was expressed in the intestine, intestinal-rectal valve, rectal gland cells, and excretory pore cells (Fig. 6B), consistent with previous reporter gene assays (Hurd, 2018). Expression of TBA-7 was also significantly lower than TBA-1 and TBA-2 (~3% (a), ~2% (b), and ~2% (c) in Fig. 2). TBB-6 was expressed in unidentified cells in the head (Fig. 1 and Fig. 6C) and the expression level was low (~4% (a), ~5% (b), and ~3% (c) of TBB-2 in Fig. 2D). TBA-8 was undetectable in the adult stage, but in second larval stage (L2) and fourth larval stage (L4) larvae, two linear signals along the anteroposterior axis were observed that correspond to seam cells in the lateral epithelium, which is consistent with a previous reporter assay (Portman and Emmons, 2004) (Fig. 1 and Fig. 6D). The expression level of TBA-8 was also significantly lower than that of TBA-1 and TBA-2 (~4% (a), ~2% (b), and ~6% (c) in Fig. 2).

## Discussion

**Fig. 7:**
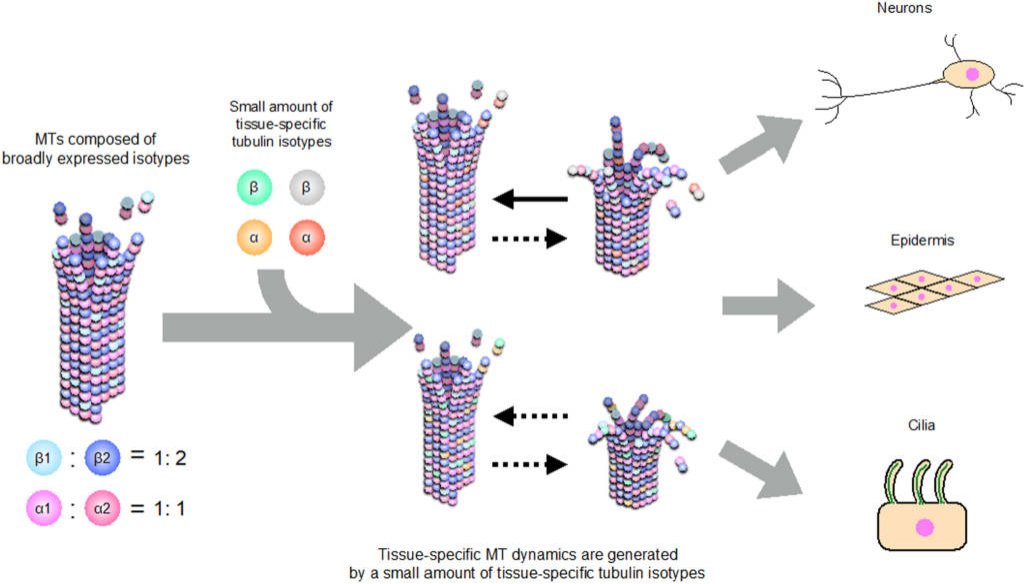
A model of the contribution of tissue-specific isotypes to tissue-specific MT properties. In early embryos, MTs are composed of four tubulin isotypes, TBA-1 (α1), TBA-2 (α2), TBB-1 (β1), and TBB-2 (β2). The ratio of α1 to α2 is about 1:1, whereas that of β1 to β2 is about 1:2 (Honda *et al.*, 2017). During development, tissue-specific tubulin isotypes are expressed at low levels and are incorporated into MTs. These small amounts of tissue-specific isotypes might modify MT properties in each tissue.

To our knowledge, this is the first report to systematically analyze the expression levels of all tubulin isotypes in a single metazoan organism. We found that the broadly expressed tubulin isotypes were expressed at significantly higher levels than tissue-specific isotypes, suggesting that the incorporation of even a small amount of tissue-specific tubulin isotypes into the MTs, which comprise mainly the broadly expressed isotypes, may confer the specific MT features of that particular tissue (Fig. 7).

Although the expression patterns of tubulin isotypes detected in these strains were generally consistent with previous studies that involved extrachromosomal arrays, GFP::TBA-6 in our study was not detected in HSN motor neurons as previously reported (Hurd *et al.*, 2010). This discrepancy could have several explanations. First, transgenes used in previous studies might contain incomplete promoter regions that are not sufficient to reproduce endogenous expression patterns. Second, some endogenous-level GFP signals in the knock-in strains may be too weak to detect, whereas GFP signals in the multicopy-transgenic strains (e.g., Hurd *et al.*, 2010, Hao *et al.*, 2011) might be expressed at a higher, and thus detectable, level.

qPCR analyses have also been used to determine transcript levels of tubulin isotypes in *C. elegans* PLM neurons (Lockhead *et al.*, 2016). In that study, transcripts of the mechanosensory neuron-specific α-tubulin isotype MEC-12 and β-tubulin isotype MEC-7 were detected at higher levels than those of the broadly expressed isotypes TBA-1 and TBB-2, whereas we found that GFP-tagged endogenous TBA-1 and TBB-2 were detected at higher levels than those of MEC-7 and MEC-12. This discrepancy might reflect differences in translational efficiency of the mRNAs of each tubulin isotype. Post-transcriptional regulation of tubulin isotype genes needs to be explored in the future.

Our collection of GFP knock-in strains also revealed the preference and efficiency with which each tubulin isotype was incorporated into different types of MTs (e.g., spindle MTs, ciliary MTs, or axonal MTs). It is unclear whether the presence of some isotypes in neuronal cell bodies was due to an excess of tubulin proteins that were not incorporated into MTs in axons or dendrites or was due to their being required in the cell bodies.

The C terminus of tubulins represents the most divergent region of these proteins and is subjected to PTMs, which can modulate MT properties. For example, detyrosination of tubulins affects mechanotransduction in skeletal and heart muscles in mice, and this disruption causes muscular dystrophy (Kerr *et al.*, 2015). Knockdown of the tubulin glycine ligase TTLL-3 results in shortened cilia in zebrafish and *Tetrahymena* *thermophila* (Wloga *et al.*, 2009). However, how isotype-specific PTMs affect MT properties *in vivo* is not well understood. Both ciliated neuron-specific α-tubulin isotype TBA-6 and the relative levels of tubulin glutamylase TTLL-11 and deglutamylase CCPP-1 are crucial for the structure and function of cilia in *C. elegans* neurons, although TBA-6 does not have polyglutamylation sites (Silva *et al.*, 2017, O’Hagan *et al.*, 2017). Thus, other tubulin isotypes expressed in the ciliary neurons, possibly TBA-1, TBA-2, TBB-1, TBB-2, and/or TBB-4, might be regulated by polyglutamylation. How PTMs in each isotype contribute to the properties of tissue-specific MTs is an important topic to be analyzed.

In other organisms, some tubulin isotypes are not replaceable by other isotypes. In *Drosophila melanogaster*, the somatic β-tubulin isotype β3 does not complement the function of the testis-specific β-tubulin isotype β2 (Fackenthal *et al.*, 1993; Hoyle and Raff, 1990). In mice, platelets require β-tubulin isotype β1 and α-tubulin isotype α4 for the assembly of a MT structure called the marginal band that maintains platelet structure (Schwer *et al.*, 2001; Strassel *et al.*, 2019). Our analysis demonstrated that TBA-5, TBA-6, and TBA-9 are expressed in distinct subsets of ciliated neurons, and these isotypes affect ciliary structures (Hao *et al.*, 2011; O’Hagan *et al.*, 2017). It will be of interest to determine whether the loss-of-function phenotype of these isotypes in *C. elegans* can be rescued by other ciliary neuron-specific isotypes, which will help us to further understand the functional specificity of tubulin isotypes.

The combination of tubulin isotypes and PTMs is proposed to generate “tubulin codes,” which can be read out by the interaction between MTs and MAPs including motor proteins (Gadadhar *et al.*, 2017). *In vitro*, MT dynamics and stability are modulated according to the composition of different human β-tubulin isotypes (Ti *et al.*, 2018; Vemu *et al.*, 2017). Thus, the combination of tubulin isotypes can fine-tune MT functions via isotype-specific PTMs and interactions with MAPs and motors (Sirajuddin *et al.*, 2014). Our analysis of the tissue-specific composition of tubulin isotypes will be a starting point for decoding tubulin codes *in vivo*, and this comprehensive collection of GFP-knock-in strains will be useful for further studies.

## Materials and Methods

### Worm strains and maintenance

Bristol N2 strain was used as wild type. Strains used and constructed in this study are listed in Table SI. All worms were grown in standard nematode growth medium (NGM), fed OP50, and kept at 20°C or 24.5°C as indicated (Table SI) (Brenner, 1974).

### Worm strain construction

The *gfp::tba-1*, *gfp::tba-2*, *gfp::tbb-1*, and *gfp::tbb-2* strains were constructed in our previous study (Honda *et al.*, 2017). The other strains were constructed using the method developed by Dickinson *et al.* (2015). For the loss-of-function strains, the GFP coding sequence and self-excision cassette (SEC) were inserted just before the start codon of each tubulin coding region; these strains show the Rol phenotype because of the mutated *sqt-1* gene in the SEC. For the GFP-fusion strains, SEC was excised by heat shock so that the GFP coding region became adjacent to the tubulin coding region (the resulting strains become non-Rol).

Each repair template fragment was cloned into the pDD282 vector (Addgene #66823) by Gibson assembly method (Gibson *et al.*, 2009). Primers used in this study are listed in Table SII. As left homology arms, 500- to 800-base pair (bp) DNA fragments upstream of the start codon of each tubulin gene were PCR-amplified from N2 genomic DNA or fosmids. As right homology arms, 500- to 3000-bp DNA fragments downstream of each stop codon were amplified. To prevent the repair template from being cleaved by Cas9, silent mutations were incorporated in the single-guide RNA (sgRNA) binding site or protospacer adjacent motif (PAM) sequence. All Cas9 target sites were chosen using the online design tool CRISPRdirect (http://crispr.dbcls.jp/) (Naito *et al.*, 2015).

To construct the *gfp::tba-4*, *gfp::tbb-4*, *gfp::tbb-6*, *gfp::ben-1*, and *gfp::mec-7* strains, purified Cas9 protein and synthesized sgRNAs were injected into the N2 worms with the respective repair template plasmid as described (Honda *et al.*, 2017). The sgRNAs were synthesized using template oligonucleotides (Chen *et al.*, 2013; Ward, 2015), the sequences for which are shown in Table SIII.

The *gfp::tba-5*, *gfp::tba-6*, *gfp::tba-7*, *gfp::tba-8*, *gfp::tba-9*, and *gfp::mec-12* strains were constructed by the plasmid-based sgRNA and Cas9 expression method (Dickinson *et al.*, 2013, 2015). For the sgRNA (F+E)-expressing parental vector, pTK73 was used as described (Obinata *et al.*, 2018). Primers are listed in Table SIV. Each primer pair was annealed and ligated to *BsaI*-digested pTK73 as described (Arribere *et al.*, 2014). The respective repair template plasmids, sgRNA vector (modified pTK73), and Cas9 vector (pDD162; *Peft-3::Cas9*, Addgene #47549; Dickinson *et al.*, 2013) were co-injected into N2 worms with three *mCherry* markers: pCFJ90, *Pmyo-2*::*mCherry* (Addgene #19327); pCFJ104, *Pmyo-3*::*mCherry* (Addgene #19328); pGH8, *Prab-3*::*mCherry* (Addgene #193595) (Frøkjaer-Jensen *et al.*, 2008).

All constructed alleles were confirmed by PCR and sequencing of the corresponding genomic regions.

### Microscopy

For live imaging of the GFP signals in the whole body of adult hermaphrodites, worms were immobilized with 0.5% phenoxypropanol on 2% agarose pads. Images were taken with a CSU-X1 spinning-disk confocal system (Yokogawa Electric, Musashino, Japan) mounted on an IX71 inverted microscope (Olympus, Tokyo, Japan) with a UPlanSApo 60×/1.30 NA silicone objective lens (Olympus) under the control of MetaMorph software (Molecular Devices, Sunnyvale, CA). All images were taken by an Orca-R2 12-bit/16-bit cooled CCD camera (Hamamatsu Photonics, Hamamatsu, Japan). Images were acquired for 40 *z*-sections with 1-μm steps at every field of view by using a 2000-ms exposure time with camera gain set to 0 and without binning. Images were processed and analyzed by ImageJ/Fiji software (National Institutes of Health, Bethesda, MD). For Fig. 1, *z*-Sectioned image stacks were projected using the Max intensity algorithm, connected to generate the image of the whole body with the MosaicJ plug-in, and then corrected to create the image of a straightened worm with the Straighten plug-in in ImageJ. Some images in Fig. 1 were enhanced according to the signal intensity using the Brightness/Contrast function in ImageJ.

Live imaging of embryos was performed as described (Toya *et al.*, 2010). In brief, hermaphrodite adults were dissected transversely with an injection needle, and embryos were collected in egg buffer (NaCl 94.4 mM, KCl 32 mM, MgCl_2_ 2.72 mM, CaCl_2_ 2.72 mM, HEPES [pH 7.4] 4 mM) (Edgar, 1995) on a cover glass. Embryos were mounted on 2% agarose pads with egg buffer and sealed with Vaseline to fill the gap between the cover glass and glass slide. GFP and mCherry images were acquired every minute at each of 21 *z*-sections with 1-μm steps and a 500-ms exposure time with camera gain of 0 and without binning by confocal microscopy as described above.

### Quantification of expression levels of each tubulin isotype

Expression levels of GFP-tagged tubulin isotypes in Fig. 2 and Fig. 3 were quantified with ImageJ/Fiji software (National Institutes of Health, Bethesda, MD). Cell types expressing GFP-labeled tubulin isotypes were identified based on WormAtlas (Altun and Hall, 2005). The signal intensities of GFP-tagged tubulins were quantified in the head region, middle body region, cell bodies, and cilia using the *z*-sectioned image stacks (the same set used in Fig. 1) projected with the Sum intensity algorithm. The expression level in the head and middle body regions was measured by the signal intensities of lines residing on worms with the Prot Profile function included in ImageJ/Fiji software. For the comparison of expression levels in cell bodies, all detectable cell body regions were selected manually, and their signal intensities were measured. For the subcellular distribution of ciliated neuron-specific isotypes, GFP signals in these neurons were measured from 5 μm outside the ciliary structure to the cell body with Prot Profile.

### Immunostaining

Embryos were collected from hermaphrodite adults of N2 and of the GFP-expressing mutant strains as described above and were incubated in egg buffer for 6–8 hours until they reached the 1.5- to 3-fold stage. These embryos were transferred to poly-l-lysine-coated slides and were fixed by conventional freeze-cracking and methanol-acetone treatment (–20°C methanol for 10 min, –20°C acetone for 5 min) (Albertson, 1984). The samples were rehydrated by passing the slide through an acetone series (90%, 70%, 50%, and 30%), followed by transfer into PBS+0.5% (w/v) Tween 20 (PBST). After incubation for 1 hour at 4°C in a humid chamber with PBST containing 0.1% bovine serum albumin, the samples were stained with rat polyclonal anti-GFP (1:200 dilution; Nacalai Tesque, Kyoto, Japan; 04404-84) and anti-mCherry (1:500 dilution; Sigma-Aldrich, St Louis, MO, USA; T9026-100UL). Secondary antibodies, Alexa Fluor 488-conjugated goat ant-rat IgG (H+L) (Invitrogen, Carlsbad, CA, USA; A-11006) at a 1:400 dilution or Alexa Fluor 568-conjugated goat anti-mouse IgG (H+L) (Invitrogen, Carlsbad, CA, USA; A-18684) at a 1:400 dilution, were incubated with the samples for 2 hours at room temperature. After immunostaining, DAPI (Dojindo laboratories, Kumamoto, Japan) was added to a final concentration of 1 μg/mL, and the samples were mounted with Prolong Diamond (Thermo Fisher Scientific, Waltham, MA, USA) for microscopy.

## Figures and Tables

**Table I:** Summary of expression patterns and knock-out phenotypes of all tubulin isotypes

Isotype		Site of expression (tissues or cells) [reference(s)]	Viability/lethality in this study	Previously reported mutant phenotype
α	*tba-1*	**epidermis**, germline, **intestine**, **muscle**, neurons [1^a^,2^b^]	viable	not detected [1^f^], neuronal malformation [3^g^], semidominant embryonic lethal [4^e^]
	*tba-2*	**epidermis**, germline, **intestine**, **muscle**, neurons [1^a^,5^b^]	viable	not detected [1^f^]
	*tba-4*	**neurons, epidermis, muscle**	viable	defects in the movement of the distal tip cell [6^f^], slow post-embryonic growth [7^f^]
	*tba-5*	ciliated sensory neurons (ADF, ADL, AFD, ASE, ASG, ASH, ASI, ASK, AWA, AWB, PHA, PHB) [8^b^]	viable	ciliary malformation [8^e^]
	*tba-6*	ciliated sensory neurons IL1 or IL2 (IL, ILD, ILV) and motor neuron (PDE) [9^c^]	viable	defects in MT ultrastructure in cilia [10^g^], behavioral abnormality of mating in males [9^g^]
	*tba-7*	intestine, excretory pore cell, intestinal-rectal valve (vir), rectal gland cells (**rect_D, rect_VL/R**) [11^c^]	viable	not detected [11^f^], abnormal neurite growth [12^e^]
	*tba-8*	seam cells [11^c^,13^c^]	viable	not reported
	*tba-9*	ciliated sensory neuron (CEPV, CEPD, and other neurons) [9^c^]	viable	behavioral abnormality of mating in males and the disrupted distribution of TBB-4 in cilia [9^g^]
	*mec-12*	mechanosensory neurons, other neurons [14^d^]	viable	abnormal mechanosensation, defects in MT structures in touch receptor neurons [15^e^]
β	*tbb-1*	**epidermis**, germline, **intestine**, **muscle**, neurons [1^a^]	viable	not detected [1^a^]
	*tbb-2*	**epidermis**, germline, **intestine**, **muscle**, neurons [1^a^]	partial embryonic lethality	spindle positioning defects [16^e^], partial embryonic lethality [1^a^]
	*tbb-4*	ciliated sensory neurons [8^b^]	viable	ciliary malformation [8^e^]
	*tbb-6*	unidentified cells	viable	not detected [11^f,g^]
	*ben-1*	multiple neurons [11^c^]	viable	insensitive to benzimidazole [17^g^]
	*mec-7*	mechanosensory neuron [18^d^,19^d^]	viable	defects in MT structures in touch receptor neurons [20^e^,21^g^]

Tissues or cells in bold indicate newly identified tissue/cell types from this study. The relevant reference(s) and methods are as indicated below.

^a^ GFP knock-in; ^b^ Transgene (translational fusion); ^c^ Transgene (transcriptional fusion); ^d^ *In situ* immunofluorescence; ^e^ Missense mutation; ^f^ RNAi; ^g^ Deletion

References: 1. Honda *et al.*, 2017; 2. Fukushige *et al.*, 1995; 3. Baran *et al.*, 2010; 4. O’Rourke *et al.*, 2011; 5. Fukushige *et al.*, 1993; 6. Cram *et al.*, 2006; 7. Simmer *et al.*, 2003; 8. Hao *et al.*, 2011; 9. Hurd *et al.*, 2010; 10. Silva *et al.*, 2017; 11. Hurd, 2018; 12. Zheng *et al.*, 2017; 13. Portman and Emmons, 2004; 14. Fukushige *et al.*, 1999; 15. Chalfie and Au, 1989; 16. Ellis *et al.*, 2004; 17. Driscoll *et al.*, 1989; 18. Hamelin *et al.*, 1992; 19. Mitani *et al.*, 1993; 20. Chalfie and Thomson, 1982; 21. Savage *et al.*, 1989.

## Abbreviations

MT: microtubule
CFEOM3: congenital fibrosis of the extraocular muscles type 3
SEC: self-excising cassette
L2: second larval stage
L4: fourth larval stage
PTM: post-translational modification
MAP: microtubule-associated protein

## Supplementary Material

Table SI

Table SII

Table SIII

Table SIV
