# Supplementary material for: Expression Patterns and Levels of All Tubulin Isotypes Analyzed in GFP Knock-In C. elegans Strains
Source: Cell Struct Funct. 2021 May 8;46(1):51–64. doi: 10.1247/csf.21022 (PMC10511039; doi:10.1247/csf.21022)
Supplement: Supplementary file 1 — Table SI [file csf_46_21022_1.pdf]

Table SI. STRAIN LIST

Strain	Genotype	Reference	Culture condition
N2	Wild type		20°C
SA250	tjIs54[pie-1promoter-gfp::tbb-2; pie-1promoter-2xmCherry::tbg-1; unc-119+]; tjIs57[pie-1 promoter-mCherry::H2B (his-48); unc-119+]	(Toya *et al.*, 2011)	24.5°C
SA854	*tbb-2* (*tj26* [*gfp::tbb-2*]) III	(Y. Honda *et al.*, 2017)	20℃
SA884	*tbb-1* (*tj30* [*gfp::tbb-1*]) III	(Y. Honda *et al.*, 2017)	20℃
SA1014	*tba-2* (*tj36* [*gfp::SEC::3×FLAG::tba-2*]) I	(Y. Honda *et al.*, 2017)	20℃
SA1016	*tba-2* (*tj38* [*gfp::3×FLAG::tba-2*]) I	(Y. Honda *et al.*, 2017)	24.5℃
SA1066	*tba-1* (*tj43* [*gfp::SEC::3×FLAG::tba-1*]) I	(Y. Honda *et al.*, 2017)	20℃
SA1067	*tba-1* (*tj44* [*gfp::3×FLAG::tba-1*]) I	(Y. Honda *et al.*, 2017)	24.5℃
SA1068	*tbb-4* (*tj45* [*gfp::SEC::3×FLAG::tbb-4*]) II	This study	20℃
SA1069	*mec-7* (*tj46* [*gfp::SEC::3×FLAG::mec-7*]) X	This study	20℃
SA1170	*tba-4* (*tj62* [*gfp::SEC::3×FLAG::tba-4*]) II	This study	20℃
SA1171	*tba-4* (*tj63* [*gfp::3×FLAG::tba-4*]) II	This study	20℃
SA1174	*tba-7* (*tj64* [*gfp::SEC::3×FLAG::tba-7*] III	This study	20℃
SA1176	*tba-7* (*tj66* [*gfp::3×FLAG::tba-7*]) III	This study	20℃
SA1178	*mec-12* (*tj68* [*gfp::SEC::3×FLAG::mec-12*]) III	This study	20℃
SA1180	*mec-12* (*tj70* [*gfp::3×FLAG::mec-12*]) III	This study	20℃
SA1204	*tbb-4* (*tj73* [*gfp::SEC::3×FLAG::tbb-4*]) X	This study	20℃

SA1205	*tbb-4* (*tj74* [*gfp::3×FLAG::tbb-4*]) X	This study	20℃
SA1207	*tbb-6* (*tj76* [*gfp::SEC::3×FLAG::tbb-6*]) V	This study	20℃
SA1208	*mec-7* (*tj77* [*gfp::3×FLAG::mec-7*]) X	This study	20℃
SA1280	*tbb-6* (*tj80* [*gfp::3×FLAG::tbb-6*]) V	This study	20℃
SA1302	*tba-9* (*tj99* [*gfp::SEC::3×FLAG::tba-9*]) X	This study	20℃
SA1303	*tba-9* (*tj100* [*gfp::3×FLAG::tba-9*]) X	This study	20℃
SA1357	*tba-5* (*tj101* [*gfp::SEC::3×FLAG::tba-5*]) II	This study	20℃
SA1358	*tba-5* (*tj102* [*gfp::3×FLAG::tba-5*]) II	This study	20℃
SA1377	*ben-1* (*tj86* [*gfp::SEC::3×FLAG::ben-1*]) III	This study	20℃
SA1378	*ben-1* (*tj87* [*gfp::3×FLAG::ben-1*]) III	This study	20℃
SA1384	*tba-6* (*tj103* [*gfp::SEC::3×FLAG::tba-6*]) I	This study	20℃
SA1386	*tba-6* (*tj105* [*gfp::3×FLAG::tba-6*]) I	This study	20℃
SA1429	*tba-8* (*tj107* [*gfp::SEC::3×FLAG::tba-8*]) X	This study	20℃
SA1430	*tba-8* (*tj108* [*gfp::3×FLAG::tba-8*]) X	This study	20℃
