# Supplementary material for: Expression Patterns and Levels of All Tubulin Isotypes Analyzed in GFP Knock-In C. elegans Strains
Source: Cell Struct Funct. 2021 May 8;46(1):51–64. doi: 10.1247/csf.21022 (PMC10511039; doi:10.1247/csf.21022)
Supplement: Supplementary file 2 — Table SII [file csf_46_21022_2.pdf]

Table SII. PRIMERS FOR HOMOLOGOUS REPAIR TEMPLATES

Allele	Template	PCR-amplified sequence	Sequence (5' to 3')[a]
tba-4 (tj63 [gfp::tba-4])	PCR-amplified N2 genomic DNA fragment including the *tba-4* locus	Left arm	F: acgacggccagtcgccggcaTTTGCAGCCCATCCCTGTTC
			R: tccagtgaacaattcttctcctttactcatCTCACGCATGGTTTTTTTCTCTG
		Right arm with a silent mutation in D218 (GAC to GAT)	F1: cgtgattacaaggatgacgatgacaagagaATGCGTGAGGTAATCTCCATTC
			R1: TCGGTCG**A**TCAACATCCAAA
			F2: TTTGGATGTTGA**T**CGACCGAGTTACACC
			R2: tcacacaggaaacagctatgaccatgttatAAACTTGTAGTCCAGGCGGG
tba-5 (tj102 [gfp::tba-5])	WRM064bA10	Left arm	F: acgacggccagtcgccggcaTTTTTAATTTTGCACGTAGAAAGTGAAAAATGG
			R: tccagtgaacaattcttctcctttactcatTTTTCCATTTGGAGCCATGG
		Right arm with a silent mutation in G10 (GGC to GGA)	F: gattacaaggatgacgatgacaagagaATGCGTGAAATAGTTTCGATTCATATC GG**A**CAAGCCGG
			R: aacagctatgaccatgttatTAGCTAATTTGGCAAACTTGGCGAAATTTGG
tba-6 (tj105 [gfp::tba-6])	WRM0614aH05	Left arm	F: cgacggccagtcgccggcaAACAATCATAGATTTCCAATATCG
			R: gtgaacaattcttctcctttactcatTGTTCGTGTTCACAACAAGTAC
		Right arm with silent mutations in P2 (CCA to CCG), Q3 (CAA to CAG), and Y4 (TAC to TAT)	F: gattacaaggatgacgatgacaagagaATGCC**G**CA**G**TA**T**AAAGGAAGTGTAAG
			R: aggaaacagctatgaccatgttatTGAACTCCTGCTTGACCCAC
tba-7 (tj66 [gfp::tba-7])	WRM0614bC10	Left arm	F: acgacggccagtcgccggcaAACCCTTGAGATGTCCGCTG
			R: tccagtgaacaattcttctcctttactcatTTCTCTCATAGTTCACTGTACTG
		Right arm with a silent mutation in I7 (ATC to ATT)	F: aggatgacgatgacaagagaATGAGAGAAGTAATCTCAAT**T**CACGTG
			R: tcacacaggaaacagctatgaccatgttatGCTGACTTTTCGAAGGCGTC

tba-8 (tj108 [gfp::tba-8])	WRM0611F04	Left arm	F: cgacggccagtcgccggcaTACAGAAAAAAAAAATCACCGAAAGACTAC
			R: gaacaattcttctcctttactcatGTTTTCGCGGATTTCGGCTC
		Right arm with a silent mutation in L73 (TTG to CTT), P2 (CCT to CCA) and S3 (TCG to AGC)	F1: aaggatgacgatgacaagagaATGCCTTCGGATGGTGTAAG
			R1: CAGTTGGTTC**A**A**G**ATCCACATAAATC
			F2: GATTTATGTGGAT**C**T**T**GAACCAACTG
			R2: ggaaacagctatgaccatgttatATTTGACAACAAAATTGGACTGAAG
			F3: caaggatgacgatgacaagagaATGCC**AAGC**GATGGTGTAAGTCAATTTTATTTATTTC
			R3: GAAATAAATAAAATTGACTTACACCATC**GCTT**GGCATtctcttgtcatcgtcatccttg
tba-9 (tj100 [gfp::tba-9])	WRM063aF04	Left arm	F: acgacggccagtcgccggcaACGGCAATGGGGTACTCAAG
			R: tccagtgaacaattcttctcctttactcatTCTGAAGAATTCGAAGAAGTAATAGC
		Right arm with a silent mutation in L75 (TTG to CTC)	F1: gattacaaggatgacgatgacaagagaATGGTCAACAATCGCTCGGTAAG
			R1: CGGCTC**G**A**G**ATCGACCATTATAG
			F2: TAATGGTCGAT**C**T**C**GAGCCG
			F2: tcacacaggaaacagctatgaccatgttatGGCTGTCTCATTGCGGTTTG

mec-12 (tj70 [gfp::mec-12])	PCR-amplified N2 genomic DNA fragment that includes the *mec-12* locus	Left arm	F: acgacggccagtcgccggcaCACACCCTGGCATGTACCTT
			R: tccagtgaacaattcttctcctttactcatTTTGCAAAAGAGGAGCTACAAG
		Right arm with silent mutation in L26 (CTG to CTT)	F1: cgtgattacaaggatgacgatgacaagagaATGGTGGGTTTTCTAGATTTTCTGAAC
			R1: CCGTGCTC**A**AGGCAGTAGAG
			F2: CTCTACTGCCT**T**GAGCACGG
			R2: tcacacaggaaacagctatgaccatgttatGCGTCTCTTTCTCTCCCACG
tbb-4 (tj74 [gfp::tbb-4])	WRM066bH01	Left arm	F: acgacggccagtcgccggcaCGTGAGCAGCAAAAAGGAAGG
			R: tccagtgaacaattcttctcctttactcatACGCATTTCTGAAATTTAATCATATTATTTC
		Right arm with silent mutation in T33 (ACC to ACA)	F1:gtgattacaaggatgacgatgacaagagaATGCGTGAAATTGTTCATATCCAGG
			R1: CTCCATTGTATGCTCC**T**GTGGGATCGATC
			F2: CCCAC**A**GGAGCATACAATGGA
			R2: cacaggaaacagctatgaccatgttatCATAACCTCCCCCGTAACCG
tbb-6 (tj80 [gfp::tbb-6])	WRM0638aC04	Left arm	F: acgacggccagtcgccggcaCCCGTTACTGCTCAGAAAAGC
			R: tccagtgaacaattcttctcctttactcatTGTTCTCTGCAATTGAGGG
		Right arm with silent mutation in S49 (TCC to TCA) and S42 (TCC to TCA)	F1: gtgattacaaggatgacgatgacaagagaATGAAAGAAATTATTAACGTTCAAGTTGG
			R1: CCATCTCCTTATAATA**T**GAAGTTATACGTTCAAGCTGGG
			F2: CTTC**A**TATTATAAGGAGATGGAAGGTGAGGG
			R2: cacaggaaacagctatgaccatgttatCATGAGATGGGGCATGGAGAG
			F3: gtgattacaaggatgacgatgacaagagaATGAAAGAAATTATTAACGTTCAAGTTGG
			R3: CGTTCAAGCTG**T**GAACCATTATCTCC
			F4: GGTTC**A**CAGCTTGAACGTATAACTTCC
			R4: cacaggaaacagctatgaccatgttatCATGAGATGGGGCATGGAGAG

ben-1 (tj87 [gfp::ben-1])	WRM0641bB11	Left arm	F: acgacggccagtcgccggcaCCCCGCCTATTTCGAAACAAC
			R: tccagtgaacaattcttctcctttactcatTTTTTCCAAGTTCTTTTTGAAGT
		Right arm with silent mutation in I30 (ATC to ATT) and S25 (TCC to TCA)	F1: gtgattacaaggatgacgatgacaagagaATGAGAGAAATTGTTCACGTTCAAGC
			R1: CATCAGGCTG**A**ATCCCATGCTCATCGGATATCAC
			F2: GCATGGGAT**T**CAGCCTGATGGAACTTATAAGGG
			R2: cacaggaaacagctatgaccatgttatGAGTGACAGACATCCGGGAC
			F3: gtgattacaaggatgacgatgacaagagaATGAGAGAAATTGTTCACGTTCAAGC
			R3: CCCATGCTCATC**T**GATATCACTTCCC-
			F4: GGGAAGTGATATC**A**GATGAGCATGGG
			R4: cacaggaaacagctatgaccatgttatGAGTGACAGACATCCGGGAC
mec-7 (tj77 [gfp::mec-7])	WRM0639bD04	Left arm	F: acgacggccagtcgccggcaGCTGCAAAACGCTGGTACTC
			R: tccagtgaacaattcttctcctttactcatGTTGCTTGAAATTTGGACCCG
		Right arm with silent mutation in G34 (GGC to GGT)	F1: gtgattacaaggatgacgatgacaagagaATGCGCGAGATCGTTCATATTC
			R1: CCCACATACTG**A**CCAGATGGGTCAATGCCG
			F2: CCCATCTGG**T**CAGTATGTGGGAGACTCTGATC
			R2: cacaggaaacagctatgaccatgttatCAAGCTGGTGGACAGAGAGGG

[a]: F: Forward; R: Reverse; Upper-case: gene-specific sequence; Lower-case: common sequences for Gibson Assembly; Bold and underline: silent mutation to make the fragment resistant to the cleavage by Cas9/sgRNA.
