# Supplementary material for: Expression Patterns and Levels of All Tubulin Isotypes Analyzed in GFP Knock-In C. elegans Strains
Source: Cell Struct Funct. 2021 May 8;46(1):51–64. doi: 10.1247/csf.21022 (PMC10511039; doi:10.1247/csf.21022)
Supplement: Supplementary file 3 — Table SIII [file csf_46_21022_3.pdf]

**Table SIII.** PRIMERS OF TEMPLATE OLIGONUCLEOTIDES FOR SGRNA SYNTHESIS

| Target | Primer sequence (5’ to 3’)[a] |
|---|---|
| Forward primers | |
| *tba-4* | gaaat**taatacgactcactatagg**TGAGATTGGTGTAACTCGGTgtttaagagctatgctggaa |
| *tbb-4* | gaaat**taatacgactcactatagg**AGTCTCCATTGTATGCTCgttttagagctagaaatagc |
| *tbb-6_1* | gaaat**taatacgactcactatagg**AAGTTATACGTTCAAGCTgttttagagctagaaatagc |
| *tbb-6_2* | gaaat**taatacgactcactatagg**ACCTTCCATCTCCTTATAATgtttaagagctatgctggaa |
| *ben-1_1* | gaaat**taatacgactcactatagg**AGGCTGGATCCCATGCTCATgttttagagctagaaatagc |
| *ben-1_2* | gaaat**taatacgactcactatagg**CCTTATAAGTTCCATCAGGCgtttaagagctatgctggaa |
| *mec-7* | gaaat**taatacgactcactatagg**ATCAGAGTCTCCCACATACgttttagagctagaaatagc |
| Reverse primers | |
| *tbb-4*, *tbb-6_1*, *ben-1_1* and *mec-7* | aaaagcaccgactcggtgccactttttcaagttgataacggactagccttattttaacttgctatttctagctctaaaac |
| *tba-4*, *tbb-6_2*, *ben-1_2* | aaaagcaccgactcggtgccactttttcaagttgataacggactagccttatttaaacttgctatgctgtttccagcatagctcttaaac |

[a]: Bold: T7 polymerase binding site; Upper-case: gene-specific sequence; Lower-case: common sequence of sgRNA scaffold; Underlined nucleotides: overlapping sequence between gene-specific forward primer and common reverse primer.
