# Supplementary material for: Expression Patterns and Levels of All Tubulin Isotypes Analyzed in GFP Knock-In C. elegans Strains
Source: Cell Struct Funct. 2021 May 8;46(1):51–64. doi: 10.1247/csf.21022 (PMC10511039; doi:10.1247/csf.21022)
Supplement: Supplementary file 4 — Table SIV [file csf_46_21022_4.pdf]

Table SIV. PRIMERS FOR CONSTRUCTING SGRNA-EXPRESSING VECTOR PTK73 SERIES

Target	Primer sequence (5' to 3')[a]
tba-5	Forward: tcttgTCCGATTTGAACACCGGCT
	Reverse: aaacAGCCGGTGTTCAAATCGGAc
tba-6	Forward: tcttgGAACAATGCCACAATACAA
	Reverse: aaacTTGTATTGTGGCATTGTTCc
tba-7	Forward: tcttgAACTCCCGCCTGCCCCACG
	Reverse: aaacCGTGGGGCAGGCGGGAGTTc
tba-8	Forward: tcttGATTGACTTACACCATCCGA
	Reverse: aaacTCGGATGGTGTAAGTCAATc
tba-9	Forward: tcttgCGCGCTATAATGGTCGATT
	Reverse: aaacAATCGACCATTATAGCGCGc
mec-12	Forward: tcttgTGCTGGGAGCTCTACTGCC
	Reverse: aaacGGCAGTAGAGCTCCCAGCAc

[a]: Upper-case nucleotides: gene-specific sequence; Lower-case nucleotides: complementary oligonucleotides with *BsaI*-digested vector
