# Supplementary figures and images for: Expression Patterns and Levels of All Tubulin Isotypes Analyzed in GFP Knock-In C. elegans Strains
Source: Cell Struct Funct. 2021 May 8;46(1):51–64. doi: 10.1247/csf.21022 (PMC10511039; doi:10.1247/csf.21022)

Fig. S1

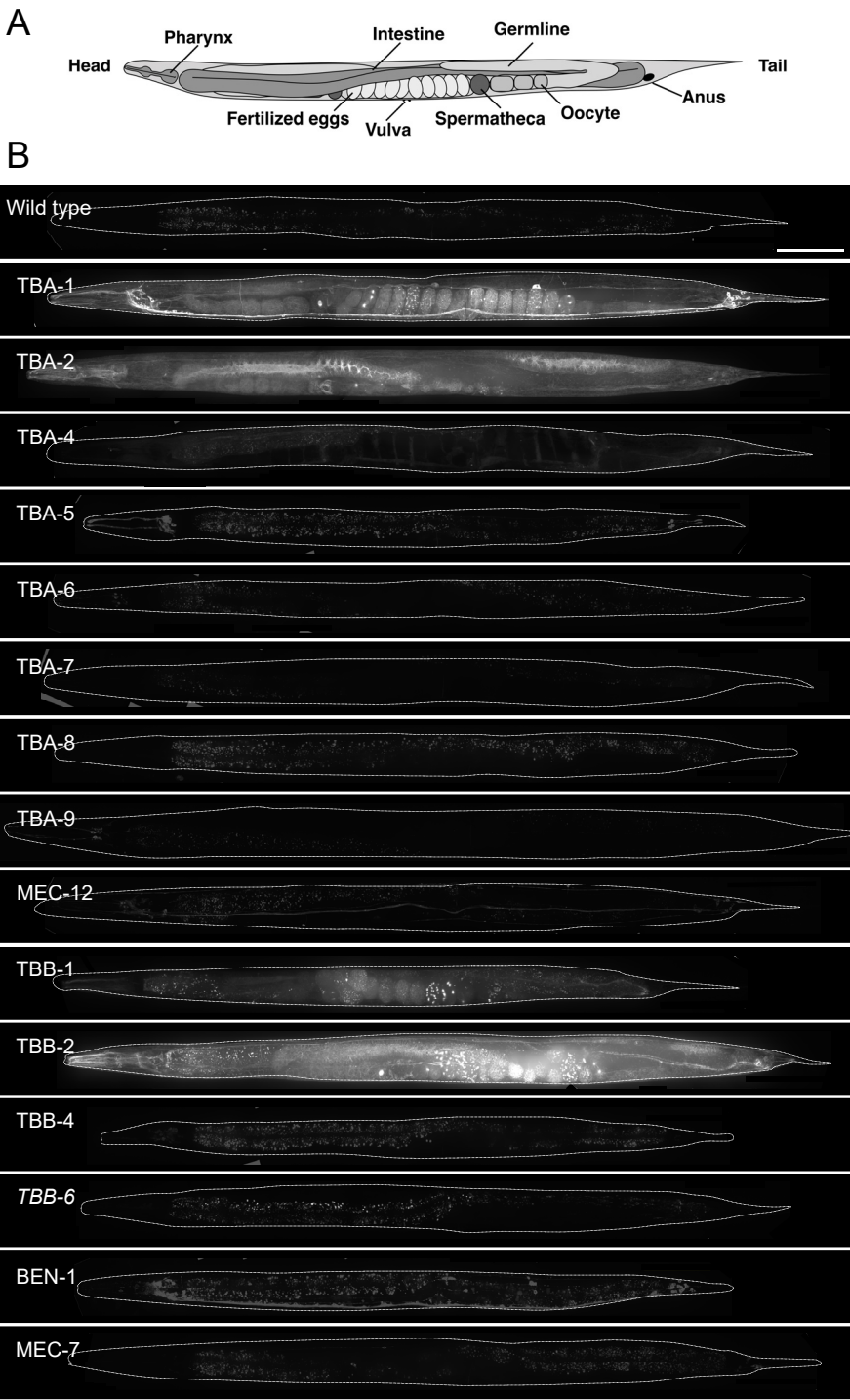

Supplement: Supplementary file 5 — Fig. S1 [file csf_46_21022_5.pdf]
